# SG-APSIC1181: Design of a temperature and humidity alert system

**DOI:** 10.1017/ash.2023.110

**Published:** 2023-03-16

**Authors:** Nanthipha Sirijindadirat

**Affiliations:** President, Central Sterilizing Services Association, Bankok, Thailand

## Abstract

**Objectives:** The central sterile supply department (CSSD) is responsible for sterilization processes, and instruments are then stored in a clean room until use. Environmental controls, such as temperature, and relative humidity, are important in preventing the spread of infection during the sterilization process and during storage of medical devices. Data regarding temperature and relative humidity readings in the operating room, and whether instruments remain covered for the duration of the operation, are difficult to obtain. For easy access to relative temperature and humidity data covering all operational intervals, a rapid and convenient notification system was designed using a software application to send temperature and humidity information to the ThingSpeak website. We identified abnormal values for temperature and relative humidity and submitted revisions for all operating periods to facilitate monitoring of the refrigeration system. **Methods:** We implemented 3 programs: (1) DHT11 humidity and temperature sensor; (2) NodeMCU ESP8266; and (3) Program Arduino IDE. **Results:** The ESP8266 board connected using WIFI SUTH Mobile, and the DHT11 displayed temperature and humidity. The temperature and humidity data were sent to the website every 10 minutes. When an alarm occurred, it triggered immediate notification via the software application. **Conclusions:** We designed a temperature and humidity alert system using DHT11. Environmental control was possible using ESP8266, and alerts were triggered in the software application when an anomaly occurred. Data were uploaded to ThingSpeak every 10 minutes. The triple system actually sends alerts through the application and records data every 10 minutes. This system can measure environmental conditions in real time.

